# P-1049. Leveraging the Consolidated Framework for Implementation Research to Appraise Policies and Practices in the Management of Emergently-Placed Central Lines

**DOI:** 10.1093/ofid/ofaf695.1244

**Published:** 2026-01-11

**Authors:** Stephanie Stroever, Joshua Preator, Madison Kranz, Lauren Dodson, Brady Miller, Megan McCoy, Trey Morris

**Affiliations:** Texas Tech University Health Sciences Center School of Medicine, Lubbock, Texas; Texas Tech University Health Sciences Center School of Medicine, Lubbock, Texas; Texas Tech University Health Sciences Center School of Medicine, Lubbock, Texas; Texas Tech University Health Sciences Center School of Medicine, Lubbock, Texas; Texas Tech University Health Sciences Center School of Medicine, Lubbock, Texas; Texas Tech University Health Sciences Center School of Medicine, Lubbock, Texas; Texas Tech University Health Sciences Center School of Medicine, Lubbock, Texas

## Abstract

**Background:**

Time is one of the most valuable resources for the critically ill or injured patient. Sterile barrier precautions may be sacrificed in emergent situations. The Healthcare Infection Control Practice Advisory Council suggests replacing or removing these lines as soon as possible. Our objective was to qualitatively assess the extent to which this guideline has been integrated into policy and practice.

**Methods:**

We conducted semi-structured interviews of physicians, healthcare epidemiologists, and infection preventionists via the SHEA Research Network. We used a blend of inductive coding and deductive application of the Consolidated Framework for Implementation Research for analysis.

**Results:**

We completed 20 interviews and achieved content sufficiency. The guideline did not require extensive supporting evidence for implementation; it was considered intuitive and a best practice. However, structural characteristics of the inner setting did not support hospital-wide policies for management of emergently placed lines. Most practices were based on unit-level policy or physician preference. We identified communication as one of the most important structural characteristics of implementation, with physical flags and verbal communication more salient than using the EMR. The implementation culture prioritizes the patient (recipient-centeredness) and requires relational connectedness across units and disciplines. Nurses tend to be the implementation leads while physicians are the guideline deliverers. Lastly, few infection prevention teams contribute to implementation in real-time. Rather, non-sterile placement is generally identified after infection. This may be due to a limitation in human resources.

**Conclusion:**

The study provided important insight into policy and practice of non-sterilely placed central lines. Prevention teams facilitate implementation by reviewing unit-level practices, fostering and/or advocating for a healthy implementation climate, and assisting with process monitoring. Strong relational characteristics and communication are critical for the protection of these high-risk patients, and healthcare teams should consider the feasibility of using communication methods external to the EMR to ensure highest fidelity to the guideline.

**Disclosures:**

All Authors: No reported disclosures

